# Organ-Specific Splice Variants of Aquaporin Water Channel AgAQP1 in the Malaria Vector *Anopheles gambiae*

**DOI:** 10.1371/journal.pone.0075888

**Published:** 2013-09-16

**Authors:** Hitoshi Tsujimoto, Kun Liu, Paul J. Linser, Peter Agre, Jason L. Rasgon

**Affiliations:** 1 Department of Entomology, Center for Infectious Disease Dynamics and the Huck Institutes of the Life Sciences, Pennsylvania State University, University Park, Pennsylvania, United States of America; 2 Malaria Research Institute and Department of Molecular Microbiology and Immunology, Bloomberg School of Public Health, Johns Hopkins University, Baltimore, Maryland, United States of America; 3 University of Florida, Whitney Laboratory, Gainesville, Florida, United States of America; Kansas State University, United States of America

## Abstract

**Background:**

Aquaporin (AQP) water channels are important for water homeostasis in all organisms. Malaria transmission is dependent on *Anopheles* mosquitoes. Water balance is a major factor influencing mosquito survival, which may indirectly affect pathogen transmission.

**Methodology/Principal Findings:**

We obtained full-length mRNA sequences for *Anopheles gambiae* aquaporin 1 (AgAQP1) and identified two splice variants for the gene. *In* *vitro* expression analysis showed that both variants transported water and were inhibited by Hg^2+^. One splice variant (AgAQP1A) was exclusively expressed in adult female ovaries indicating a function in mosquito reproduction. The other splice variant (AgAQP1B) was expressed in the midgut, malpighian tubules and the head in adult mosquitoes. Immunolabeling showed that in malpighian tubules, AgAQP1 is expressed in principal cells in the proximal portion and in stellate cells in the distal portion. Moreover, AgAQP1 is expressed in Johnston’s organ (the “ear”), which is important for courtship behavior.

**Conclusions And Significance:**

These results suggest that AgAQP1 may play roles associated with mating (courtship) and reproduction in addition to water homeostasis in this important African malaria vector.

## Introduction

Malaria is the most devastating vector-borne disease. Recent data indicate that over 250 million new cases and approximately 800,000 deaths are caused by malaria each year [1]. Although the number of cases has been reduced over the past decade, malaria remains an important health and economic burden in endemic countries. Malaria transmission occurs when an infected adult female *Anopheles* mosquito bites a human host. *Anopheles* mosquitoes must live long enough to take a second blood meal to transmit the *Plasmodium* parasites responsible for malaria. In the field, retention of water and nutrients is a critical factor affecting mosquito survival [2].

Due to their large surface area to volume ratio, regulation of water uptake and release is a major issue for insects, as their limited water reserves can be easily depleted by evaporation or excretion. During their life, mosquitoes experience significant change in their environmental water exposure. Eggs are laid on or near water, larvae and pupae are strictly aquatic, whereas adults are terrestrial. For most species, female mosquitoes require a blood meal for egg development. During blood feeding, female body mass increases by more than two times, limiting movement and increasing the probability for the mosquito to be killed by the host or predators [3]. Therefore, rapid excretion of water from the blood meal is necessary for female mosquitoes to survive to reproduce [4].

Aquaporins (AQPs) are transmembrane protein water channels found from bacteria and archaea to eukaryotes. Water molecules pass through aquaporins in a selective and efficient manner [5]. Although aquaporins are passive water channels, they can facilitate water movement by differential expression or translocation in response to appropriate stimuli [6]. Two major classes of aquaporins have been recognized: one is water-specific and the other allows passage of water and small molecules such as glycerol or urea (aquaglyceroporins). AQPs typically consist of a 28 kDa polypeptide with six transmembrane domains and connecting loops (A-E). The signature motifs of aquaporins are asparagine-proline-alanine (NPA) residues in loops B and E, which are strictly conserved. NPA motif-containing loops are folded into the pore to form the interior hourglass shape, which limits the movement of protons and selectively allows passage of water molecules by their dipole interaction [7]. The water-specific aquaporins contain conserved arginine/aromatic (R/ar) residues that form a constriction in the pore to repel molecules other than water [8].

In the Dengue vector mosquito, *Aedes aegypti*, AeaAQP (DRIP—*Drosophila melanogaster* CG9023 and AGAP008842 ortholog) is expressed in tracheolar cells in Malpighian tubules and has an implied function in respiration [9]. Recently, Drake et al. showed not only AeaAQP (AaAQP1 in ref [9]) but also other aquaporins are important for diuresis in *Ae* *. aegypti* [10]. We previously described an aquaporin (AgAQP1: AGAP008843, CG7777 ortholog) in the mosquito *Anopheles gambiae*. AgAQP1 was detected in stellate cells within the Malpighian tubules by immunofluorescence. Mosquitoes with depleted AgAQP1 expression exhibited increased desiccation resistance, indicating the importance of AgAQP1 in water homeostasis [11].

Among the many aquaporins described to date, the occurrence of splice variants has been reported in only a few. For example, mammalian AQP4 consists of several splice variants that may differ in relative water permeability [12]. In this study, we report a second splice variant of this AQP gene. The second variant has similar permeability to water as the original variant when expressed in *Xenopus* oocytes. Expression analysis of the splice variants revealed that one splice form (AgAQP1A) is specifically expressed in adult female ovaries, while the other (AgAQP1B) is expressed in larvae, pupae and adults, with expression in the midgut, hindgut, Malpighian tubules and the male and female head (likely in the Johnston’s organ).

## Results

### There are two splice variants for AgAQP1

**Figure 1:**
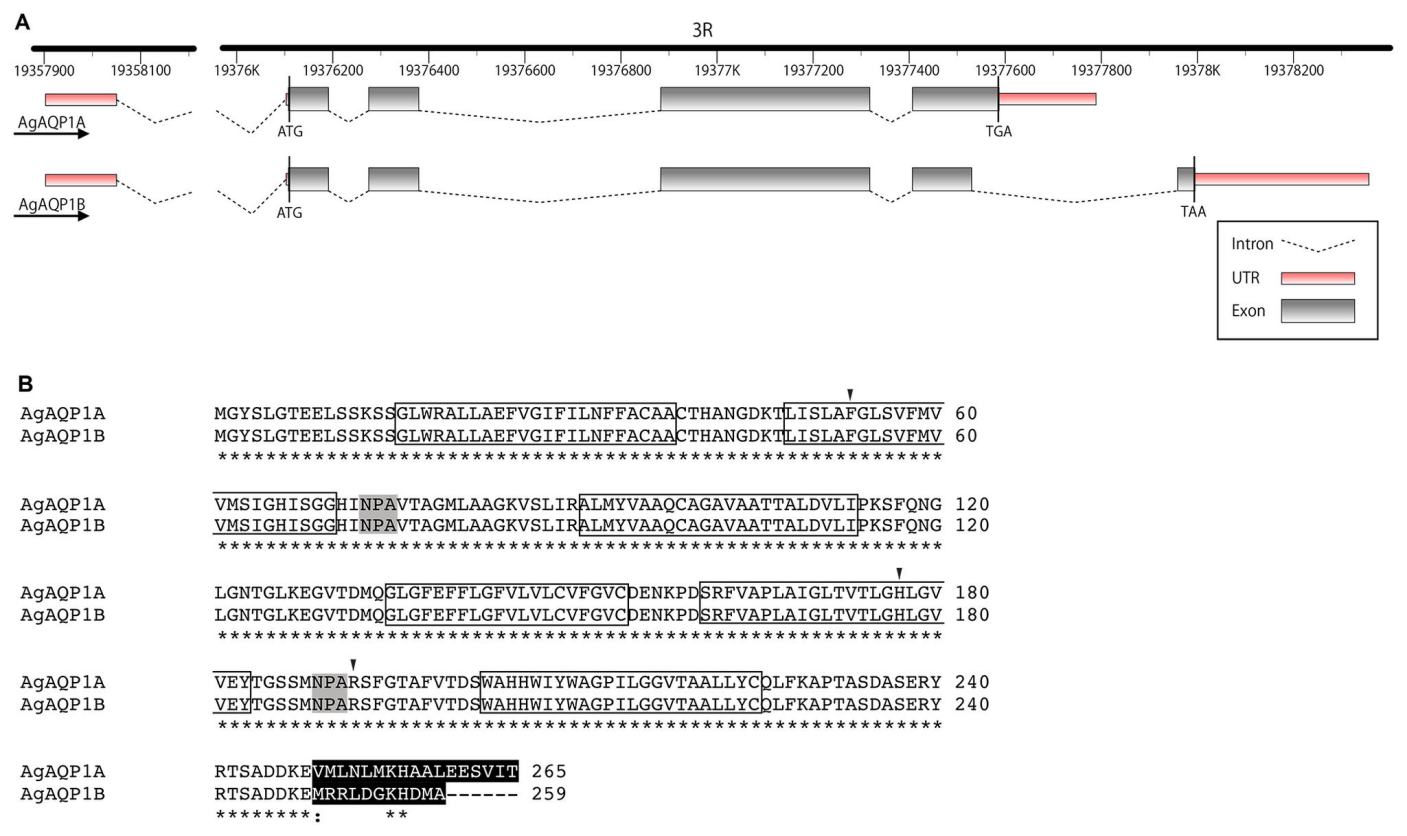
Gene and polypeptide structures of AgAQP1A and B. *A*, genetic structures of the splice variants AgAQP1A and AgAQP1B on Chromosome 3R. The structures are identical from 5’ end to the 4th intron. AgAQP1A consists of 5 exons while AgAQP1B consists of 6 exons. Untranslated regions (UTRs) are shown in red. *B*, amino-acid sequence alignment by ClustalW2 of the two variants. Predicted transmembrane domains are boxed, while NPA motifs are shaded with grey. Divergent residues at C termini are in white letters in black background. Arrowheads indicate conserved R/ar residues (F53, H177 and R192). * indicates identical residue : indicates similar residue.

Through RACE, we identified two splice variants for AgAQP1. Figure 1A shows the gene structures of the two variants on chromosome 3R. One encodes a polypeptide identical to the AgAQP1sequence we previously identified (GenBank accession number: JF342682) [11]. We now designate this variant A (AgAQP1A). Referring to the genome sequence of the *An. gambiae* PEST strain, AgAQP1A consists of 5 exons as predicted in VectorBase. The second variant encodes a polypeptide identical to “*Anopheles gambiae* aquaporin 1” (GenBank accession number: AB523397), which we designate variant B (AgAQP1B). AgAQP1B consists of 6 exons including an additional intron with canonical splice sites (GT-AG) at 3’ end of the gene. The two splice forms differ only at 3’ end of the mRNA which includes coding sequence for 17 and 11 different amino acids at the C terminal cytoplasmic tail for variants A and B, respectively (Figure 1B). Using the TMHMM server, we predicted six transmembrane domains with two NPA motifs in loop B and E for both variant A and B (Figure 1B).

### AgAQP1B is a water channel that is inhibited by Hg^2+^

**Figure 2:**
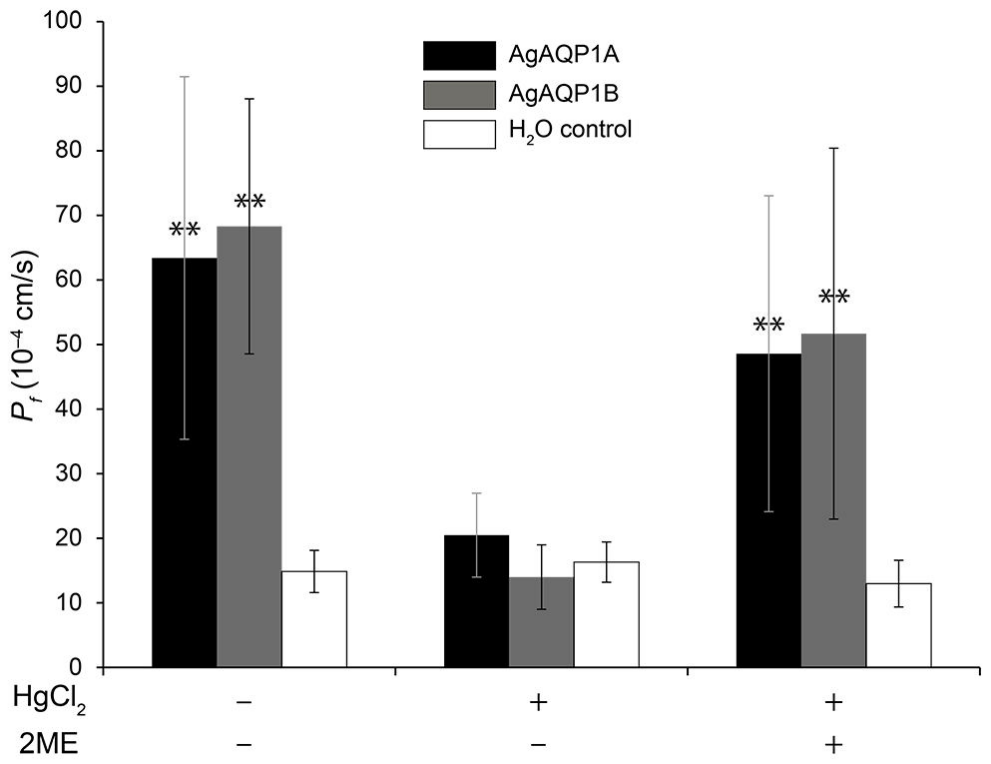
H_2_O permeability of AgAQP1A and B expressed in *Xenopus laevis* oocytes. Osmotic water permeability in 70 mOsm hypotonic solution (mean ± SD). Coefficient of osmotic water permeability (*P*_*f*_ in 10^-4^ cm/s) is shown in the *y*-axis. HgCl_2_: incubation in 0.5 mM HgCl_2_ for 5 min; 2ME: 5 mM 2-mercaptoethanol for 10 min following HgCl_2_ treatment. Statistical significance is indicated with asterisks (**: P < 0.01).

To confirm that AgAQP1B is a functional water channel, we cloned and expressed the gene in *Xenopus laevis* oocytes. We also expressed AgAQP1A for comparison. The oocyte-swelling assay indicated that AgAQP1B is a water channel that transports water as efficiently as AgAQP1A (Figure 2). Water permeability of aquaporins is inhibited by mercurial sulfhydryl reagents, which can be reversed by reducing agents [5]. Both variants of AgAQP1 were inhibited in the presence of 0.5 mM Hg^2+^, which was reversed after treatment with 5 mM 2-mercaptoethanol. Neither splice form was capable of transporting glycerol (data not shown).

### Developmental stage and organ-specific expression of the two splice variants

**Figure 3:**
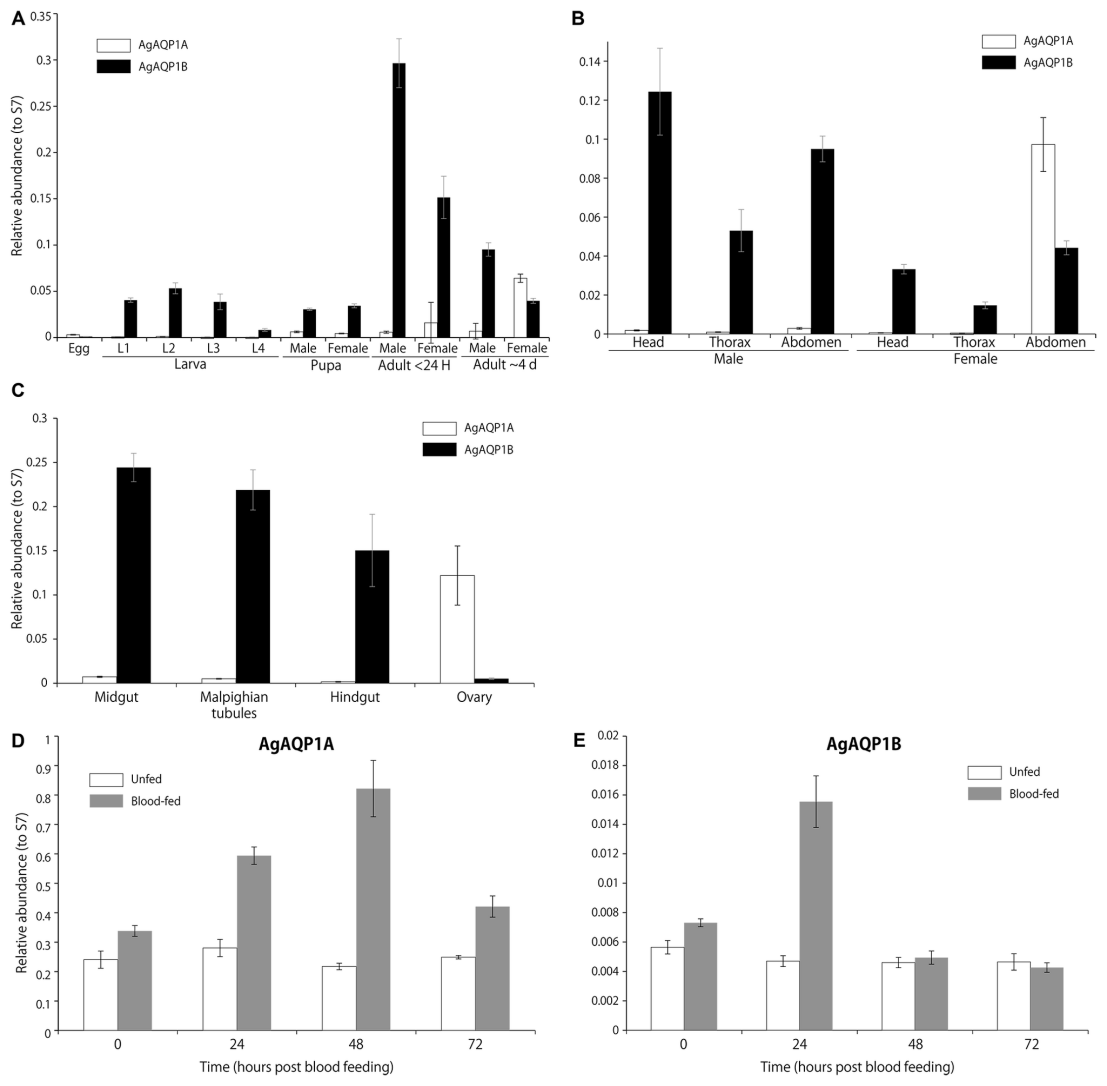
Expression profile of AgAQP1A and AgAQP1B by qRT-PCR (mean ± SD). *A*, in developmental stages, AgAQP1B is expressed in larval to adult stages with dramatic upregulation in young adults. AgAQP1A is expressed only in older adult females. *B*, AgAQP1B is expressed throughout the mature adult body, while AgAQP1A is expressed only in female abdomen. *C*, Ovaries specifically express AgAQP1A. AgAQP1A (*D*) and AgAQP1B (*E*) expression in ovaries from unfed and blood-fed mosquitoes. AgAQP1A is highly upregulated in blood-fed ovaries during egg maturation (≤ 48 h post blood feeding: pbf) returning to baseline 72 h pbf, while AgAQP1B is transiently upregulated at 24 h pbf. Note expression of AgAQP1A is much higher than AgAQP1B (see scales on vertical axes).

Using variant-specific qRT-PCR, we determined the expression profiles of AgAQP1A and B. AgAQP1B is expressed in all life stages except the egg, while AgAQP1A is expressed only in the abdomens of older adult females (Figure 3A, B). In the abdomen, AgAQP1A expression is ovary-specific while AgAQP1B is expressed in all body parts assayed (Figure 3C).

Since AgAQP1A is ovary-specific, we assessed expression of both splice forms in response to a blood meal. AgAQP1A transcript increased during egg development, returning to baseline after egg maturation (~4 fold at 48 h from 0 h post blood feeding: pbf) (Figure 3D). AgAQP1B expression transiently increased about 2-fold at 24 h pbf, although expression is very low (one-fiftieth of AgAQP1A at baseline) (Figure 3E).

### Immunofluorescence localization

**Figure 4:**
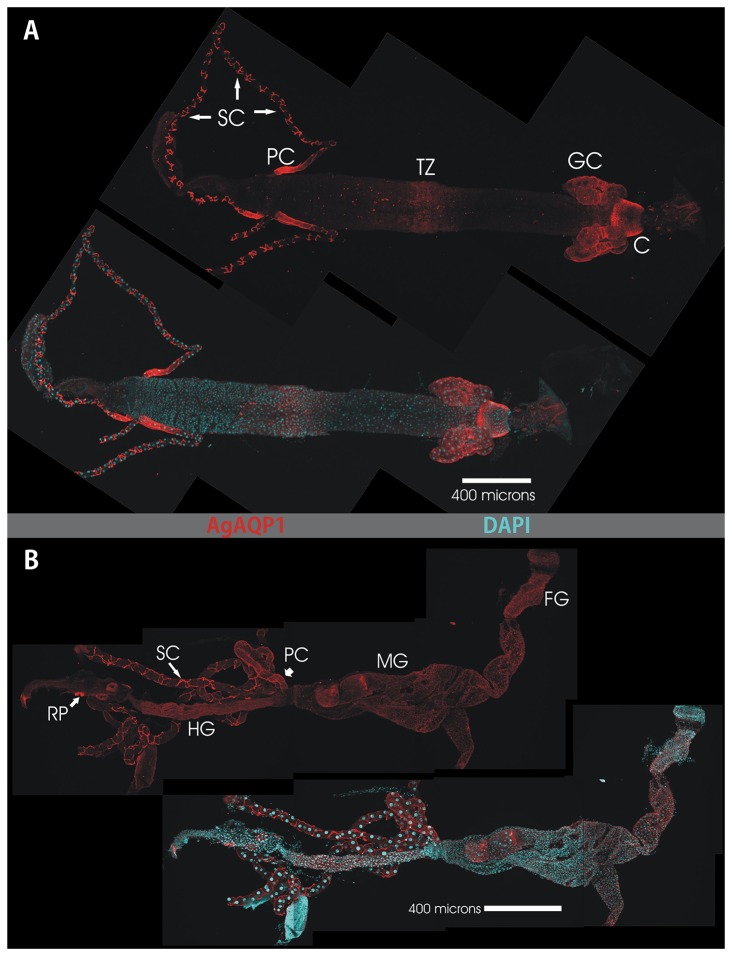
Whole-mount confocal microscopy of AgAQP1 localization in the larval and adult female alimentary canals. This figure shows two montages of maximum projection images of dissected alimentary canals from larval (*A*) and adult (*B*) *Anopheles gambiae*. Each panel shows TRITC immunofluorescence (red) for AgAQP1 localization (upper figure of each panel) and a merged image with DAPI staining of nuclear DNA (lower figure in each panel). Anterior is to the right. Prominent staining for AgAQP1 in the larval gut (panel *A*) is evident in the cells of the Cardia (labeled C), the Gastric Caecae (GC) the midgut Transitional Zone (TZ) the proximal Principal Cells of the Malpighian Tubules (PC) and the Stellate Cells (SC) of the Malpighian Tubules. In panel B, the adult alimentary canal shows areas of prominent staining in the Foregut (FG), Midgut (MG), proximal Principal Cells (PC) and Stellate Cells (SC) of the Malpighian tubules, Hindgut (HG) and the Rectal Pads (RP).

**Figure 5:**
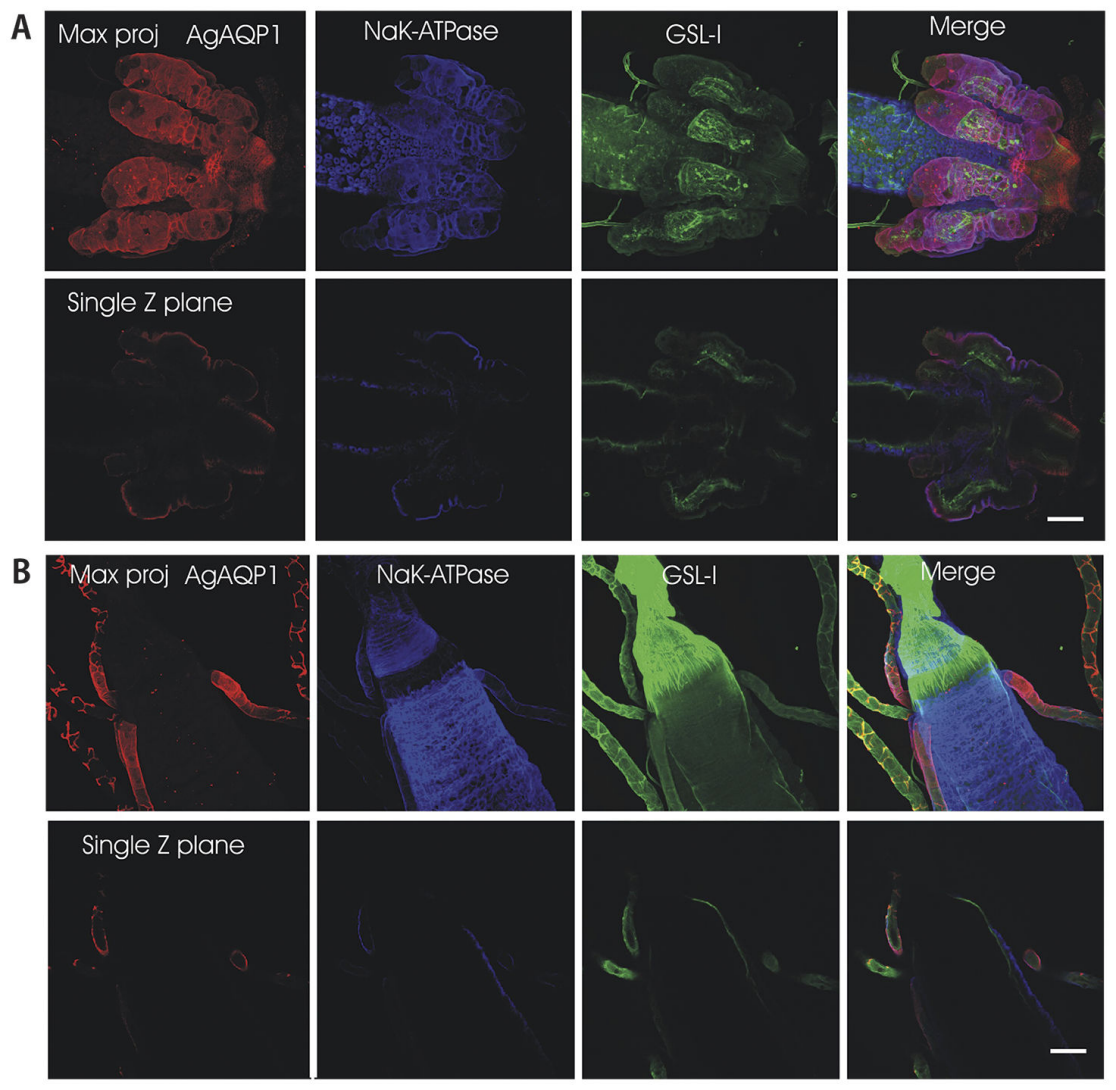
Confocal microscopy of AgAQP1 in the larval gastric caecae and Malpighian tubule area indicating basal localization. This figure shows a single larval gut at the region of the Gastric Cacae (GC) (*A*) and midgut-hindgut junction with Malpighian tubules (*B*). TRITC (red) staining indicates AgAQP1, Cy5 (blue) indicates the basal membrane marker NaK-ATPase and GSL-1 (green) indicates a plant lectin that binds to various extracellular matrices including the caecal membrane which is internal to the caecal cavity. The top row of each panel shows a maximum projection of several z-planes. Note that in the merge panel at the right end, red and blue signals on the lobes of the cacae co-localize, indicating co-localization of AgAQP1 and NaK-ATPase on the basal membranes of the caecal cells. The lower row of panels showing GC are from the same z-stack but present a single z plane. Again, note that AgAQP1 and the NaK-ATPase colocalize. Note the intense labeling for AgAQP1 in the proximal Principal Cells and the Stellate cells of the Malpighian tubules. As in the GC, AgAQP1 labeling in the Malpighian tubules is on the basal surface of both Principal cells. Scale bar 120 µm.

Our available antibody was raised against the N-terminus of AgAQP1 and is predicted to bind to both splice forms. In the larval alimentary canal AgAQP1 staining is evident in the cardia, the gastric caecae, the transitional zone of the midgut, the Malpighian tubule stellate cells, and the proximal Malpighian tubule principal cells (Figure 4A). Analysis of a single plane of the Z stack indicates that AgAQP1 is localized on the basal rather than the luminal membranes, as assessed by its colocalization with basally-located NaK-ATPase [13] (Figure 5).

**Figure 6:**
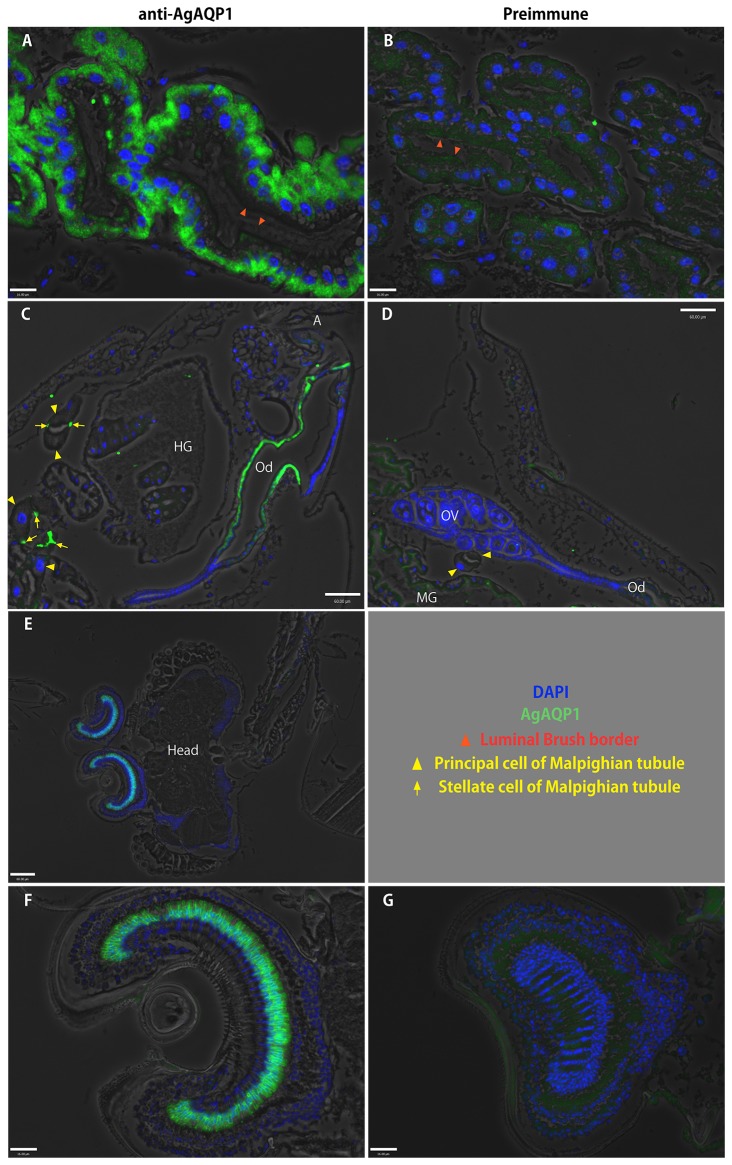
Localization of AgAQP1 in adult and larval mosquitoes by immunofluorescence of sections. Fluorescence micrograph images using anti-AgAQP1 (green). Blue: DAPI (cell nuclei). *A*, AgAQP1 is expressed on the basal side of the female midgut, (lumen indicated by red arrowheads). *B*, preimmune. *C*, AgAQP1 is expressed in oviduct (Od). Immunofluorescence appears to be in posterior portion of the oviduct (A: anus). In this figure, Malpighian tubule principal cells with large nuclei (yellow triangles) with immunolabeled stellate cells (yellow arrows) either traverse or cross section are included. Hindgut rectum with rectal pads is also seen (HG: hindgut; OV: ovary). *D*, preimmune. *E* and *F*, AgAQP1 is expressed in Johnston’s organ (JO). *G*, preimmune. Shown are the male JO. Scale bar *A*, B, F, *G*: 16 µm; C, D, *E*: 60 µm.

In the adult alimentary canal, AgAQP1 staining is evident in the foregut, midgut, hindgut, rectal pads, and Malpighian tubule stellate cells and proximal principal cells (similar to larvae) (Figure 4B). Expression in the midgut was again limited to the basal membrane rather than the luminal membrane (Figure 6A). We did not detect protein signal from oocytes and associated follicles, but rather specifically in the oviduct (Figure 6C), although the transcript of AgAQP1A was detected abundantly in the ovaries. Interestingly, we also detected AgAQP1 in the basal segment (pedicel) of antenna. This structure is called the Johnston’s organ (JO) (Figure 6E, F), which has a mechanosensory function to detect sound and is specifically important for courtship behavior [14]. Figure 5E and F show the male JO, which is larger than the female; the female JO also expresses AgAQP1 in a similar distribution (data not shown).

## Discussion

We detected two splice variants for AgAQP1, which are differentially expressed in *An. gambiae*. The structural differences between these two variants are confined to a short portion at the 3’ end that encodes the C-termini of the proteins, presumably located in the cytosolic tails. This small variation may lead to functional differences such as intracellular trafficking [15], mRNA stability [16] or translation [17].

In both larval and adult mosquitoes, we detected AgAQP1 in the stellate cells of the distal region of the Malpighian tubules (MT) as in the previous study [11], but also in the principal cells of the proximal region of the tubules (Figures 4A and 5B). It is known that in the Malpighian tubules, the proximal and distal regions are physiologically distinct [18-21]. The difference in AgAQP1 expression in principal cells between the distal and proximal regions of the MT adds to these observations and suggests that water movement may be different in different parts of the mosquito renal system. Colocalization of AgAQP1 and NaK-ATPase in larval gastric cecae and absence of NaK-ATPase in the MT also indicate the different functions of these two organs (Figure 4B).

A putative ortholog gene in *D. melanogaster* (CG7777) has three splice variants (FlyBase Genome annotation, release 3.2, 2004), whose variations are also at the 3’ end of the transcripts. Unfortunately, the function(s) of CG7777 have not been studied. Goto et al. identified 3 splice variants for the CG7777 homolog gene, BaAQP1 in the Antarctic midge, *Belgicaantarctica* , which also differ in the 3’ ends [22], suggesting that the splice variation may be ancestral in lower Diptera.

AgAQP1A and B expressed in *X. laevis* oocyte membrane showed high water permeability with a similar efficiency (Figure 3). Both isoforms showed reversible sensitivity to Hg^2+^ inhibition, although AgAQP1 lacks cysteine in the loop E, which is a known site for Hg^2+^ inhibition [23]. The impermeability of glycerol suggests that AgAQP1 is a water-specific channel, which is also evidenced by the presence of conserved R/ar residues, phenylalanine (F), histidine (H) and arginine (R) at positions 53, 177 and 192 (corresponding to F56, H180 and R195 in rat AQP1, Figure 1B), respectively [8]. Mutation of H177 to glycine did not change the permeability of glycerol, which indicated that this residue might not be important for its water/glycerol selectivity (data not shown).

We attempted to raise antibodies against C-termini of AgAQP1 to differentiate the splice variants, but these antibodies had high non-specific binding and were not useful. We successfully raised an antibody against N-terminus [11]. Using the anti-N-terminal antibody, immunofluorescence indicated that AgAQP1 is localized in baso-lateral side of the adult midgut epithelium and larval alimentary canal tissues, which suggests that there is likely another aquaporin expressed on the luminal side. For example, in the silkworm *Bombyx mori*, the water-specific AQP-Bom1 (DRIP ortholog) is specifically expressed in the apical (luminal) side of the rectal epithelial cells, whereas another water-specific AQP-Bom3 (CG7777 ortholog) is expressed in the basal membrane of the same cells [24]. Peptides of AGAP008842, another predicted aquaporin gene in *An. gambiae*, were retrieved from the adult midgut brush-border microvilli proteome [25]. Therefore, we speculate that AGAP008842 may be brush-border specific, while AgAQP1 is baso-lateral specific in the mosquito midgut.

We found that AgAQP1A is almost exclusively expressed in the ovaries of mature female adults at the transcript level, which implicates its function in female reproduction and egg development. However, we found the protein (either AgAQP1A or AgAQP1B) exclusively in the lining of oviduct, but not in ovarian follicles by immunofluorescence (Figure 6C). We attempted to knockdown AgAQP1 in the ovaries by dsRNA injection, but the transcript levels were not affected. Therefore, we can only speculate as to the function of AgAQP1A in the ovary. AgAQP1A may assist reproduction, perhaps by facilitating lubrication of the oviduct during oviposition. Alternatively, the transcript may be stored in the oocytes without translation to be used for embryonic development. qRT-PCR analysis of blood-fed mosquito ovaries showed increased AgAQP1A (throughout egg maturation), which suggests that AgAQP1A has some functions in egg maturation (Figure 3D). AgAQP1B increased transiently at early stages (24 h pbf) of egg maturation, but absolute transcript levels were very low. An orthologous gene in *Ae* *. aegypti* (AaAQP2) also increased 24 hrs pbf, which implies a possible conserved function of these orthologs in these two mosquito species [10]. Preliminary analysis of transcript level in the female reproductive system (ovarian follicles vs. oviducts) showed presence of AgAQP1A and AgAQP1B transcript in the oviduct, while follicles exclusively express AgAQP1A (not shown). Nevertheless, we did not detect transcript in embryos. Since sampled eggs were laid overnight and not finely timed, detailed analysis of AgAQP1A expression in embryonic developmental stages may reveal its function in embryogenesis. In *D. melanogaster*, transcript of the ortholog CG7777 is abundantly found in stage 1-3 embryos, but is quickly degraded, reappearing in stage 13 (Berkeley Drosophila Genome Project [26,27]). This phenomenon may be similar in *An. gambiae* and the reason for absence of transcript detection in this study.

qRT-PCR indicated that AgAQP1B is abundantly expressed in the adult head. Immunofluorescence indicated that AgAQP1 protein is localized in the Johnston’s organ (JO). The JO is located in the second basal segment of adult antennae and consists of a number of vibration-sensing units called scolopidium (*pl*. scolopidia), each with two or three sense cells. In *Ae* *. aegypti* , a male JO contains 7,000-7,500 scolopidia, which means that a single male mosquito contains ~15,000 scolopidia and ~30,000 sense cells in its paired antennae [28,29]. Recent studies indicate that hearing and adjustment of wingbeat frequency are critical for courtship behavior [13,30]. Harmonic convergence during courtship increases successful copulation [31]. RNAi knockdown of AgAQP1 in the male head was attempted, and while transcript levels were significantly reduced at some time points, results were variable between experimental replicates and there was no change in protein levels by immunofluorescence or Western blot (data not shown). Nevertheless, the presence of aquaporin in confined cells in the JO suggests that it may be important for hearing and successful completion of mating, and could be an intriguing potential target for applied mating disruption mosquito control strategies.

## Materials and Methods

### Ethics Statement

Mosquitoes were allowed to feed on anesthetized Swiss Webster mice to develop eggs for colony maintenance or for experiments as noted. Mice were purchased from the National Cancer Institute, Frederick (Frederick, MD), and were housed in the Pennsylvania State University animal care facility. Mosquito blood feeding was approved under Penn State IACUC protocol #35790.

### Mosquitoes

The *An. gambiae* Keele strain was maintained in a walk-in insectary at the Vector Genetics and Control Laboratory at the Pennsylvania State University. Larvae were reared on ground TetraMin® Tropical Flakes (Tetra Holdings Inc., Blacksburg, VA). Adults were maintained on 10% sucrose solution at 27 ± 1°C and 80 ± 5% relative humidity with a 14 h/10 h light/dark cycle.

### Expression analysis

For developmental stage analysis, eggs, larvae (first, second, third and fourth instars), male and female pupae, young (<24 hrs after eclosion) and old (~4 days after eclosion) adults were processed for RNA extraction and cDNA synthesis. For adult body part-specific and female abdominal organ-specific analysis, adults ~5 days after eclosion were used. Organ dissection was performed in PBS and dissected organs were immediately pooled in TRI reagent. For ovary-specific blood-meal response analysis, adult females (5-7 days old) were fed on an anesthetized mouse for 10-15 min. Ovaries were dissected at 0 (within 30 min), 24, 48 and 72 hrs post blood feeding (pbf). RNA extraction was performed immediately after dissection and stored at -80 °C until used.

### RNA extraction and cDNA synthesis

Whole mosquitoes or dissected organs were homogenized in TRI reagent (Applied Biosystems, Carlsbad, CA) in a 1.5 mL microcentrifuge tube. Total RNA was extracted following the manufacturer’s instructions. Extracted RNA was treated with DNase (TURBO DNAfree, Life Technologies, Carsbad, CA) for 1 hr at 37 °C. First-strand cDNA synthesis was performed on 1 µg of total RNA in 20 µL reaction using Accuscript High Fidelity Reverse Transcriptase (Agilent Technologies, Santa Clara, CA).

### Rapid Amplification of cDNA Ends (RACE)

Extracted RNA was processed using the GeneRacer Kit (Invitrogen, Carlsbad, CA) with a custom anchor oligonucleotide (Table 1) for cDNA synthesis. RNA was extracted from all life stages (egg, 1st, 2nd, 3rd, 4th instars, male and female pupae, and young [< 24 hrs after eclosion] and old [~4 days] adults of both sexes) by TRI reagent. RNA processing for RACE was performed with 3 µg RNA according to the manufacturer’s suggested protocol. cDNA was synthesized with the processed RNA and Oligo dT anchor primer using Accuscript High Fidelity Reverse Transcriptase. Gene-specific primers were designed based on the 5’ and 3’ regions of the predicted AGAP008843 in VectorBase. RACE PCR was performed with the gene-specific primers and anchor primer (Roche) or GeneRacer 5’ primer/GeneRacer 5’ nested primer (Invitrogen) (Table 1). RACE PCR products were separated by agarose gel electrophoresis, and bands excised for gel extraction with the QIAquick Gel Extraction kit (QIAGEN, Valencia, CA). Gel-extracted PCR products were cloned into pJET1.2/blunt vector using the pJET PCR Cloning Kit (Fermentas, Glen Burnie, MD). The sequences of the inserted fragments were determined by sequencing.

**Table 1:** primers used for this study.

**Primer name**	**Sequence (5’–3’**)
**For RACE work**	
Oligo dT-anchor primer*	GACCACGCGTATCGATGTCGACTTTTTTTTTTTTTTTTTTTTTTT
PCR anchor primer*	GACCACGCGTATCGATGTCGAC
GeneRacer^TM^ 5′ Primer*	CGACTGGAGCACGAGGACACTGA
GeneRacer^TM^ 5′ Nested Primer*	GGACACTGACATGGACTGAAGGAGTA
GSPAg3F1	AGCTGGGCTCATCACTGGATCTACTGGGCT
GSPAg3F2	ACTGGATCTACTGGGCTGGACCGATCCT
GSPAg3R4	TGTCCGATCGACATCACCACCATGAACAC
GSPAg3R5	CCACCATGAACACGCTCAGCCCGAAT
GSPAg3R6	AATGCCAGCGAGATCAGCGTTTTATCTCCA
**For real-time PCR**	
8843-1qPCR-F	TCCAGTACGGAAAACCCCCAAACCGA
8843-1qPCR-R	TGATGTGAAGTTGCGGTGCGGATGAGA
8843-2qPCR-F	ATGCGTCGACTGGACGGCAAGCA
8843-2qPCR-R	ACACACCACAACATTCGACGGGACACA
AGS7UC	GTGAGGTCGAGTTCAACAACAAGAA
AGS7DD	GGCACCGGCACGTAGATGA
**For *Xenopus* oocyte expression**	
8843-2F2EcoRI	ATAGAATTCCATGGGATACAGCCTAGGAACG
8843-2RXbaI	ATATCTAGATTACGCCATATCATGCTTGCC

For * refer to Materials and Methods. GSPAg3 prefixed primers are specific for 3’ (F1 and F2) and 5’ (R4, R5, and R6) of AGAP008843. GSPAg3F2, GSPAg3R5 and GSPAg3R6 are used for nested RACE PCR with PCR anchor primer or GeneRacer^TM^ 5′ Nested Primer. For real-time PCR, 8843-1 primer pair is specific against 3’ region of AgAQP1A, and 8843-2 primer pair is against 3’ AgAQP1B.

### Gene structure

FancyGene [32] was used to present gene structures on the genome. Alignments of amino-acid sequences were performed using ClustalW [33]. Prediction of transmembrane domains was performed using the TMHMM server (http://www.cbs.dtu.dk/services/TMHMM/).

### Real-time quantitative PCR (qRT-PCR)

Primers were designed against 3’ variable regions of AgAQP1A and B. cDNA was diluted to 1/50 in nuclease-free H_2_O and 2.5 µL was used for 10 µL reactions using the RotorGene Q qPCR system (QIAGEN). Reactions were performed with 5 min at 95 °C, followed by 45 cycles of 95 °C for 5 sec and 60 °C for 10 sec and melt curve analysis from 50 °C to 95 °C. Expression was calculated relative to a housekeeping gene (ribosomal protein S7) by the -2^ΔΔCt^ method [34]. Primer sequences are listed in Table 1.

### Cloning, expression in *Xenopus laevis* oocytes and swelling assay

AgAQP1A was cloned in the previous study [11]; AgAQP1B was cloned by similar methods. Briefly, coding sequence was amplified using primers containing restriction sites at the 5’ end, and after restriction digestion, PCR products were ligated into the pXβG-myc vector. The purified construct was linearized by XbaI digestion for *in vitro* complementary RNA (cRNA) transcription using T3 RNA polymerase (Agilent Technologies, Inc., Santa Clara, CA). Synthesized cRNA was purified with the RNeasy Mini kit (QIAGEN). *X. laevis* oocytes were defolliculated with collagenase I (Sigma-Aldrich, St. Louis, MO) and injected with 5 ng of cRNA or 50 nL of nuclease-free water as control. Injected oocytes were incubated for 3 days in ~200 mOsm Modified Barth’s Solution (MBS: 88 mM NaCl, 1.0 mM KCl, 2.4 mM NaHCO_3_, 15 mM Tris, 0.8 mM MgSO_4_, 0.4 mM CaCl_2_, 0.3 mM Ca(NO_3_)_2_, pH 7.6) for overexpression of aquaporin in the oocyte plasma membrane, then subjected to swelling assay [5] by transferring to MBS diluted to 70 mOsm. The change of oocyte volume was monitored at room temperature by microscopic video capture for 60 seconds and the relative volume (V/V_0_) was calculated from the cross-sectional area at the initial time (A_0_) and after a time interval (A_t_): V/V_0_ = (A_t_/A_0_)^3/2^. The coefficient of osmotic water permeability (*P*_*f*_) was determined from the initial slope of the time course [d(V/V_0_)/dt], average initial oocyte volume (V_0_ = 9 × 10^−4^ cm^3^), average initial oocyte surface area (S = 0.045 cm^2^), the molar volume of water (V_w_ = 18 cm^3^/mol), and the osmotic solute gradient (osm_in_ − osm_out_). *P*_*f*_ = (V_0_ × *d*(V/V_0_)/*dt*)/(S × V_w_ × (osm_in_ − osm_out_). A minimum of five individual oocytes were tested in each group.

To test the effect of Hg^2+^ on water permeability, oocytes were placed in 500 µM HgCl_2_ in MBS for 5 min prior to the swelling assay. To test reversibility, Hg^2+^-treated oocytes were transferred to 5 mM 2-mercaptoethanol in MBS for 10 min. Glycerol permeability was also assessed by replacing diluted MBS with MBS mixed with an equal volume of iso-osmotic (200 mOsm) glycerol solution.

### Immunofluorescence detection (whole-mount)

Fourth-instar larvae or 48-hour old adult females were cold anesthetized and dissected in primary fixative (4% p-formaldehyde in 0.1M sodium cacodylate buffer) as previously described [13]. Isolated alimentary canal tissue was incubated in primary fixative over night at 4 °C. Tissues were then washed with Tris-buffered saline (TBS), transferred to pre-incubation medium (PIM) (TBS with 0.1% Triton X-100, 2% normal goat serum and 1% bovine serum albumin) and incubated at room temperature for 2 h. Primary AgAQP1 antibody [11] was diluted in PIM (1:1000) and tissues incubated over night at 4 °C with gentle agitation. AgAQP1 antibody was raised against an N-terminus peptide and binds to both variants of AgAQP1. The following day, tissues were washed first with TBS and then with fresh PIM. Tissues were then suspended in PIM containing TRITC-conjugated goat anti-rabbit secondary antibody (Jackson Immunoresearch Laboratories, West Grove, PA; diluted 1:500) over night at 4 °C with gentle agitation. The following day, tissues were washed in TBS, incubated in TBS with DAPI and mounted on well-slides with 60% glycerol/TBS containing *p*-phenylenediamine to inhibit fluorescence quench. Samples were viewed and digital images captured with a Leica SP5 Laser Scanning Confocal Microscope. Images were assembled into figures using CorelDraw 12 software.

Immunofluorescence detection (sections): Adult mosquitoes were paraffin-embedded and sectioned. Slides containing sections were used for immunofluorescence detection using anti-AgAQP1 antibody [11]. Sections were blocked and permeabilized with 10% goat serum in PBS and 0.2% Triton X-100 at 4 °C overnight followed by incubation with anti-AgAQP1 antibody (1:300 in PBS with 10% goat serum) at 4 °C overnight and anti-rabbit IgG secondary antibody conjugated with Alexa 488 (1:1,500 in PBS with 10% goat serum). Sections were washed with PBS four times at room temperature after each step. One drop of ProLong Antifade with DAPI (Invitrogen) was placed on the section-containing slides, covered by a cover slip and sealed by nail polish. Images were obtained on a Nikon 90i upright fluorescence microscope equipped with a digital camera with Volocity imaging software (PerkinElmer, Waltham, MA).
